# Supplementary material for: Cytosolic sequestration of the vitamin D receptor as a therapeutic option for vitamin D-induced hypercalcemia
Source: Nat Commun. 2020 Dec 7;11:6249. doi: 10.1038/s41467-020-20069-4 (PMC7721737; doi:10.1038/s41467-020-20069-4)
Supplement: Supplementary file 1 — Supplementary Information [file 41467_2020_20069_MOESM1_ESM.pdf]

Cytosolic sequestration of the Vitamin D Receptor as a therapeutic option for vitamin D–induced hypercalcemia

Daniela Rovito[1-4], Anna Y. Belorusova[5], Sandra Chalhoub[1-4], Anna-Isavella Rerra[1-4], Elvire Guiot[1-4], Arnaud Molin[6,7], Agnès Linglart[7,8], Natacha Rochel[1-4], Gilles Laverny[1-4*], Daniel Metzger[1-4*].

[1] Institut de Génétique et de Biologie Moléculaire et Cellulaire, Illkirch, France.
[2] Centre National de la Recherche Scientifique, UMR7104, Illkirch, France.
[3] Institut National de la Santé et de la Recherche Médicale (INSERM), U1258, Illkirch, France.
[4] Université de Strasbourg, Illkirch, France.
[5] Medicinal Chemistry, Respiratory, Inflammation and Autoimmunity, BioPharmaceuticals R&D, AstraZeneca, Gothenburg, Sweden.
[6] Université de Normandie, UNICAEN, CHU de Caen Normandie, Service de Génétique, EA 7450 BIOTARGEN, Caen, France.
[7] Reference Center for Rare Diseases of Calcium and Phosphorus Metabolism (OSCAR), France.
[8] Université de Paris Saclay, AP-HP, Hôpital Bicêtre, DMU SEA, INSERM, U1185, Le Kremlin Bicêtre, France.
* Correspondence to Gilles Laverny (laverny@igbmc.fr) or Daniel Metzger (metzger@igbmc.fr).

Supplementary information files

- **Supplementary Figure 1 - 6**
- **Supplementary Table 1 - 4**

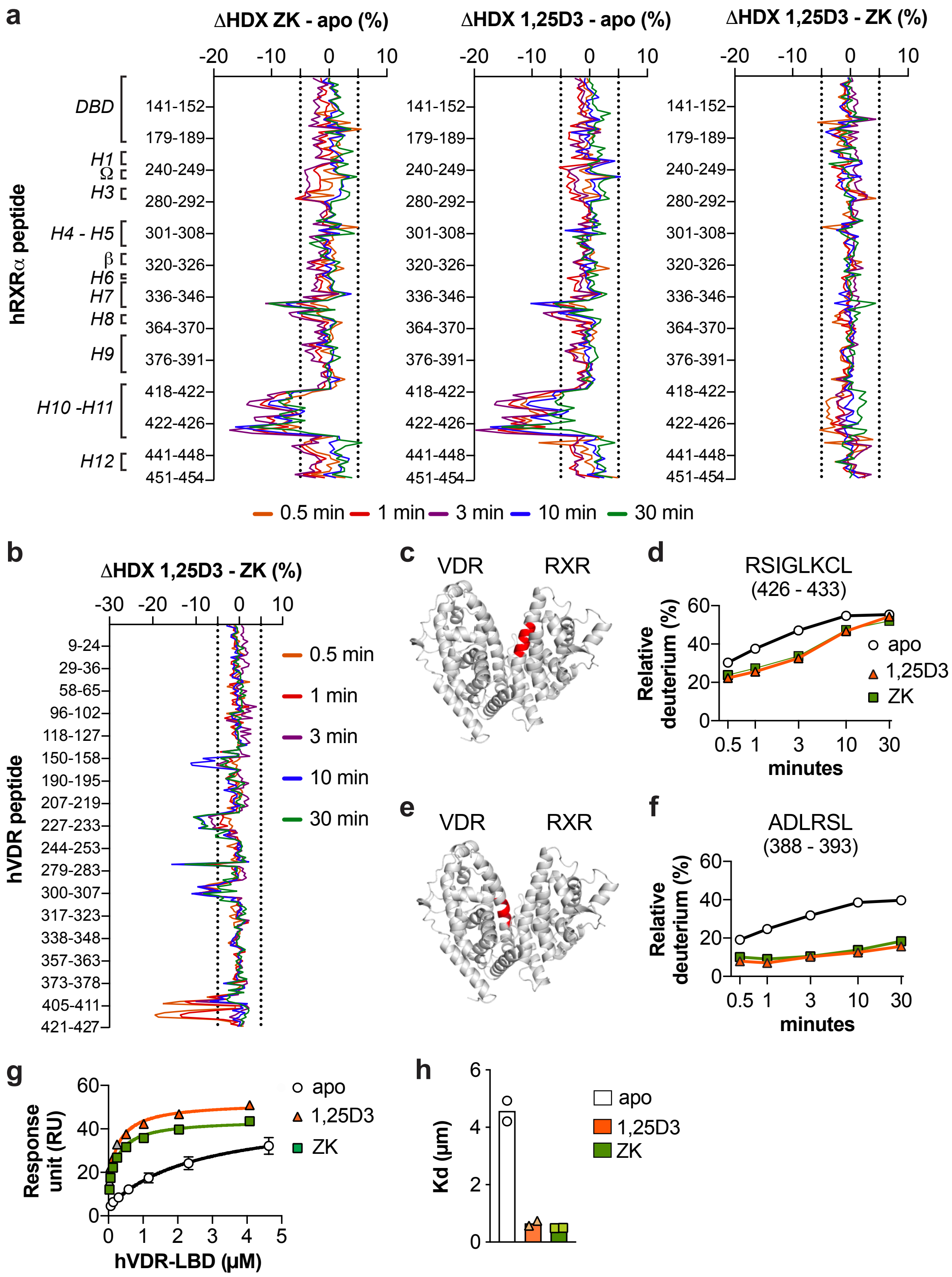

Supplementary Fig. 1. Effect of ligands on hVDR and hRXRαΔNTD conformations. (a) Differential deuterium uptake of hRXRα peptides between ZK-bound, 1,25D3-bound and apo hVDR/hRXRαΔNTD heterodimers. **(b)** Differential deuterium uptake of hVDR peptides between ZK-bound and 1,25D3-bound hVDR/hRXRαΔNTD heterodimers. Dashed lines represent a ΔHDX of 5 %. Peptides from H10 of hRXRα (426-RSIGLKCL-433) **(c)** and of hVDR (388-ADLRSL-393) **(e)** mapped onto hVDR/hRXRα LBD (PDB: 1DB1[44]), and differential deuterium uptake plots in ZK-bound, 1,25D3-bound and apo hVDR/hRXRαΔNTD heterodimers of RSIGLKCL (**d**) and ADLRSL (**f**) peptides. n = 3 technical replicates / condition. Analysis of the interactions between hRXRα-LBD and ZK-bound (ZK), 1,25D3-bound (1,25D3) and apo hVDR LBDs by surface plasmon resonance (**g**) and calculated Kd (**h**). n= 2 independent biological replicates / condition.

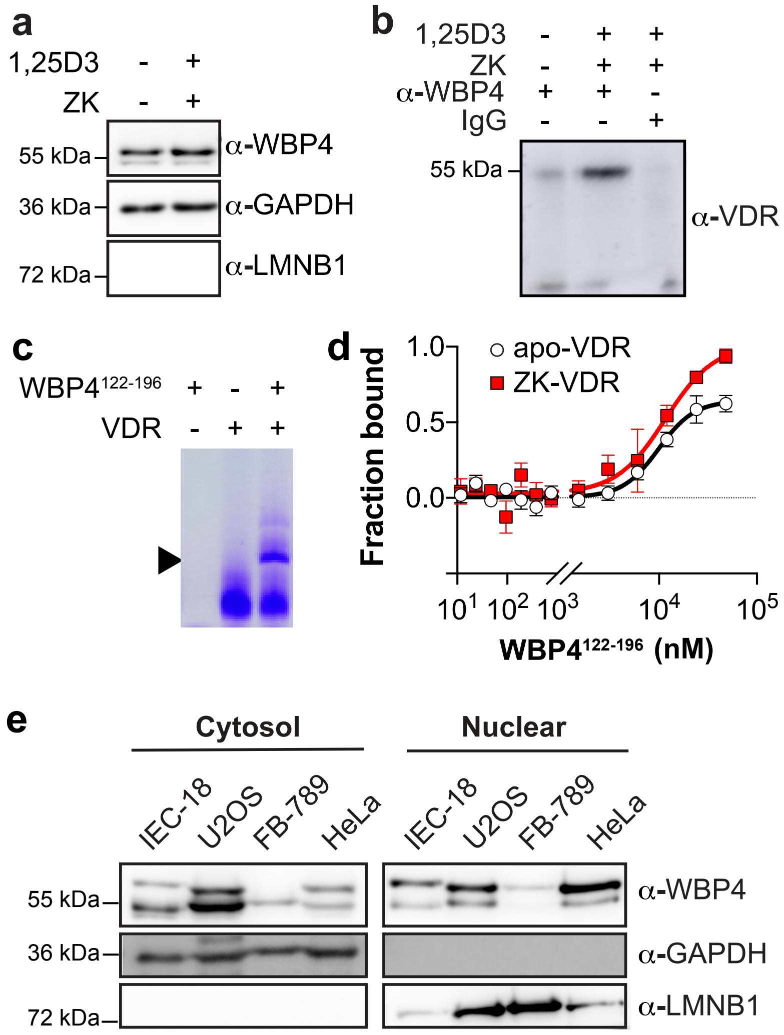

Supplementary Fig. 2. WBP4 cellular localization and characterization of its interaction with VDR. (a) Representative WBP4 immunoblot of cytosolic extracts of FB-789 cells treated for 1.5 h with vehicle, or with 100 nM 1,25D3 and 1 µM ZK. Uncropped blots in Source Data. (**b**) Representative VDR immunoblot of WBP4-immunoprecipitated cytosolic extracts of FB-789 cells treated for 1.5 h with vehicle, or 100 nM 1,25D3 and 1 µM ZK. Cytosolic extracts from FB-789 cells treated for 1.5 h with 100 nM 1,25D3 and 1 µM ZK immunoprecipitated with Rabbit IgG were used as a negative control. Uncropped blots in Source Data. **(c)** Native PAGE of hWBP4 amino acids 122 to 196 and ZK-bound hVDR full-length. Analysis by maldi MS of the band indicated by the arrow revealed the presence of the hVDR and hWBP4 polypeptides. Source data are provided as a Source data file. **(d)** WBP4 binding to hVDR measured by microscale thermophoresis. Unlabelled hWBP4 polypeptides (aa 122-196) were titrated into a fixed concentration of labelled recombinant VDR in the presence or absence (apo) of saturating concentrations of ZK. Isotherms were averaged over three consecutive measurements and fitted according to the law of mass action to yield the apparent dissociation constant. Kd=11±4 µM for ZK-bound VDR and Kd > 20 µM for apo-VDR. **(e)** Representative WBP4 immunoblot of cytosolic and nuclear extracts of IEC-18, U2OS, FB-789 and HeLa cells. GAPDH and LMNB1 were used as internal controls. Unprocessed blots in Source Data.

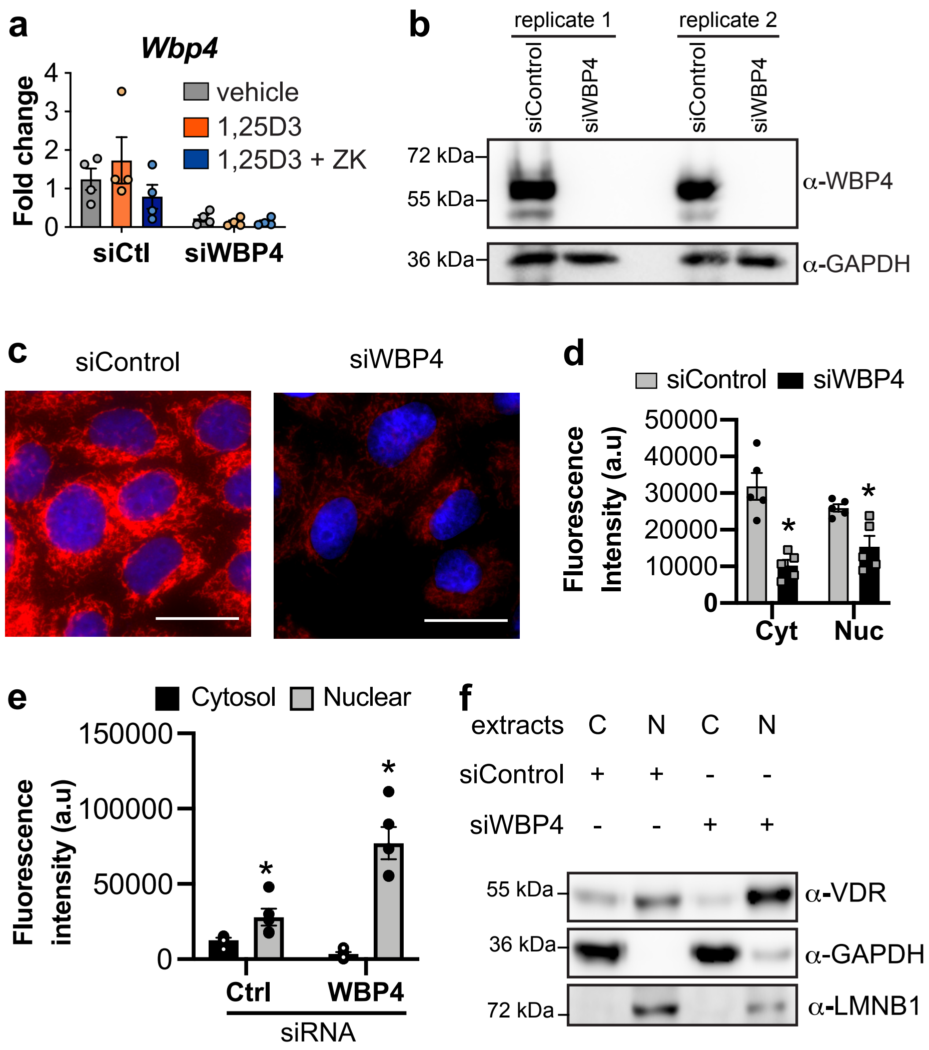

Supplementary Fig. 3. Effect of WBP4-silencing. WBP4 transcript **(a)** and protein **(b)** levels, as well as WBP4 immunocytostaining **(c)** of IEC-18 cells transfected with WBP4 siRNA or unrelated siRNA (Control, Ctl). Nuclei are stained with DAPI. Scale bar: 20 µm. Data in **(a)** represent n = 4 independent biological replicates / condition. **(d)** Quantification of the intensity per µm^2 of WBP4 immunostaining in WBP4- (siWBP4) or unrelated- (siControl) silenced IEC-18 cells. n = 5 cells / condition. **(e)** Quantification of the intensity per µm^2 of VDR immunostaining in WBP4- (siWBP4) or unrelated- (siControl) silenced IEC-18 cells of Fig. 4e. n = 5 cells / condition. **(f)** Representative VDR immunoblot in cytosolic (C) and nuclear (N) extracts from WBP4- (siWBP4) or unrelated- (siControl) silenced IEC-18 cells. GAPDH and LMNB1 were used as internal controls. Unprocessed blots in Source Data. * $p < 0.05$ vs. siControl, two-way ANOVA with Tukey post-hoc test. The exact significant p values are provided in **Supplementary Table 4**.

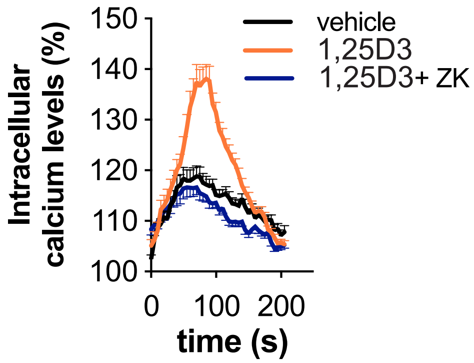

Supplementary Fig. 4. Effect of VDR ligands on intracellular calcium levels. Intracellular calcium flux in IEC-18 cells treated for 48 h with vehicle, 100 nM 1,25D3, or co-treated with 100 nM 1,25D3 and 1 μM ZK. n > 40 cells, 3 independent biological replicates / condition. Data are expressed as % of basal levels.

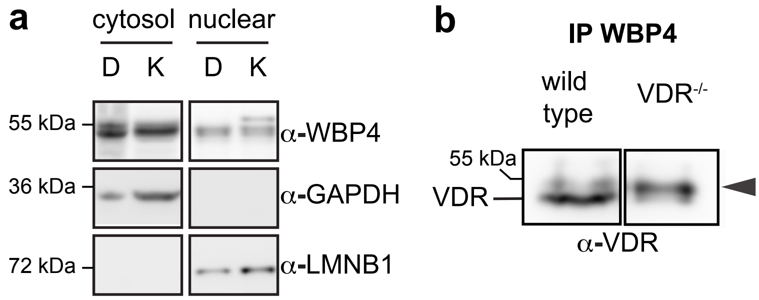

Supplementary Fig. 5. WBP4 cellular localization and interaction with VDR in mice. (a) Representative WBP4 immunoblot of cytosolic and nuclear extracts from duodenum (D) and kidney (K) of a wild type mouse. GAPDH and LMNB1 were used as internal controls. Uncropped blots in Source Data. **(b)** Representative VDR immunoblot in WBP4 immunoprecipitated renal extracts from wild type and VDR-null ($VDR^{-/-}$) mice. The arrowhead represents WBP4 antibody heavy chains. Uncropped blots in Source Data.

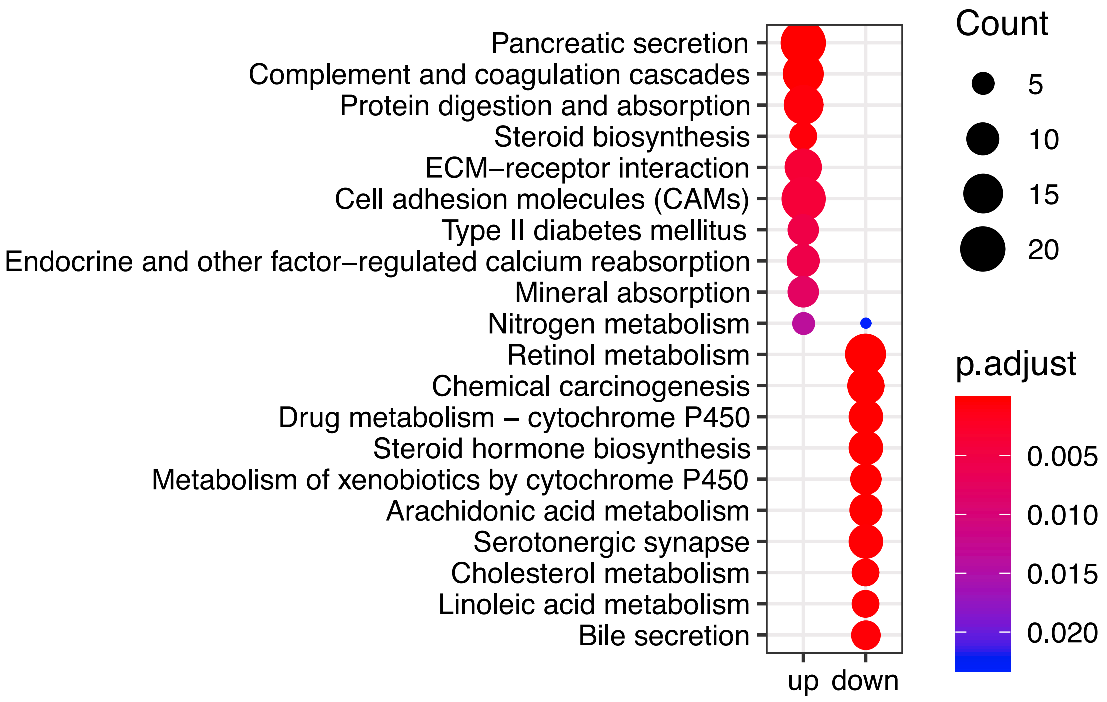

Supplementary Fig. 6. Pathways analyses of genes deregulated by 1,25D3. KEGG pathway of the genes up and down regulated in the duodenum of mice treated with 1,25D3 for 6 h. Source data are provided as a Source Data file.

Data Set	hVDR- hRXRαΔNTD
HDX reaction details	50 mM Tris, 200 mM NaCl, 2% glycerol, 1 mM TCEP, pDread = 7.6, 25 °C
HDX time course (min)	0.5, 1, 3, 10, 30
HDX control samples	Maximally-labeled control (zebrafish VDR LBD protein)
Back-exchange (mean / IQR)	48.05% / 9.69%
# of Peptides	VDR: 179; RXRα: 127
Sequence coverage	VDR: 92.1%; RXRα: 88.7%
Average peptide length / Redundancy	VDR: 7.91 / 3.99; RXRα: 8.33 / 3.98
Replicates (biological or technical)	3 (technical)
Repeatability	0.049 (average standard deviation)
Significant differences in HDX	p-value < 0.05 and ΔHDX > 5% (biological threshold)

Supplementary Table 1. HDX data summary.

#term ID	term description	gene count	FDR
GO:0043229	intracellular organelle	60	2.42E-26
GO:0005622	intracellular	63	4.25E-26
GO:0044422	organelle part	52	4.25E-26
GO:0044446	intracellular organelle part	51	5.51E-26
GO:0044424	intracellular part	62	8.99E-26
GO:0044444	cytoplasmic part	53	5.59E-25
GO:0044464	cell part	64	1.47E-24
GO:0005737	cytoplasm	56	1.24E-22
GO:0043232	intracellular non-membrane-bounded organelle	37	1.24E-22
GO:0032991	protein-containing complex	40	1.41E-21

Supplementary Table 2: Top 10 cellular component terms obtained by Gene Ontology (GO) annotation of the proteins identified by MS and selectively enriched (p value < 0.01) in VDR-immunoprecipitated cytosolic extracts of 1,25D3 and ZK co-treated IEC-18 cells compared to vehicle-treated cells.

Oligonucleotides

RT-qPCR (mouse)

Gene	Forward primer 5' - 3'	Reverse primer 5' - 3'
cyp24a1	GGCGGAAGATGTGAGGAATA	GCCCAGCACTTGGGTATTTA
slc30a10	GGTGATTCCCTGAACACCGA	ACGTGCAAAAGAACACCTCTG
slc37a2	TAGGGCCAGACTAGAGCCA	ACATGCTCATCTCTGCCGAC
nkain1	GGAGTCAAAGCCACCGATGA	CCTAGCTCTGTTCGGCTTCG
atp2b1	CACGTCCCGCGACCC	TGTATACATCTCGCCCGCAG
18 s	AGCTCACTGGCATGGCCTTC	CGCCTGCTTCACCACCTTC

qPCR (mouse)

genomic region	Forward primer 5' - 3'	Reverse primer 5' - 3'
cyp24a1_VDRE	CAGTGACTCCCGGATGAACC	TCAGGAAGGCTGCTAAAGGC
slc30a10_VDRE	ACAGGGTGCAAAGAAGCCAA	AAGGACCTCCTCCCTACGTC
slc37a2_VDRE	TCCTGGGTGTGCCGATTCTA	CCTGGTCTGGTTGTTACCGT
nkain1_VDRE	GCCTGTGCTAACCACTTCGT	TCGTTTAACACGTACCAGGCA
atp2b1_VDRE	TTAAACCCAGTAACCTGCAATGT	AAGGTCACCTGAACCTCGAAA

RT-qPCR (rat)

Gene	Forward primer 5' - 3'	Reverse primer 5' - 3'
Cyp24a1	TCCATGAGGCTTACCCCAAG	GCGTATTCACCCAGAACCGT
Atp2b1	GTGGAGGTTCCATTCAGGGG	CTCGGATACCCCACAGGAGT
S100g	GACAGCAAGCAGCACAGAAAA	TGGACAGCTGGTTTGGATCG
Trpv6	GAGCACAGGTTGTGGCTACT	CCAAGACCATACTCTCGCCC
Wbp4	CCTGGAAAGTTCTGAGGCGA	TACTCGCGGCGCCTGAC
Gapdh	AGTGCCAGCCTCGTCTCATA	GGTAACCAGGCGTCCGATAC

RT-qPCR (human)

Gene	Forward primer 5' - 3'	Reverse primer 5' - 3'
CYP24A1	GACATCCAGGCCACAGACAA	ACCACCATCTGAGGCGTATT
RPLP0	CGTCCTCGTGGAAGTGACAT	TAGTTGGACTTCCAGGTCGC

Antibodies

Target	Host species	Supplier	Reference	Dilution
VDR	Rabbit	Cell Signaling	D2K6W, #12550	1/2000
WBP4 (IP and IF)	Rabbit	Abcam	ab108144	IP: 3µg IF:1/200
WBP4 (WB)	Rabbit	Abcam	ab272629	1/1000
LMNB1	Rabbit	Abcam	ab16048	1/10000
GAPDH	Rabbit	Cell Signaling	14C10, #2118	1/20000
anti-rabbit HRP conjugated	Goat	Jackson Immunoresearch	111-035-003	1/10000
anti-rabbit CY3 conjugated	Goat	Jackson Immunoresearch	111-165-003	1/400
anti-mouse HRP conjugated	Goat	Jackson Immunoresearch	115-035-003	1/10000
anti-rabbit IgG (WB)	Mouse	Cell Signaling	L27A9, #3678	1/2000
anti-rabbit IgG (IP)	Rabbit	Diagenode	C15410206	3µg

Supplementary Table 3. Summary of the oligonucleotides and antibodies used.

Supplementary Table 4: exact p-values

Figure 1a	Tukey's multiple comparisons test	95% confidence interval	p-value
**Cyp24a1**			
	1,25D3 vs Vehicle	1.079 to 2.791	<0.0001
	ZK vs Vehicle	0.2528 to 1.965	0.0091
	1,25D3 + ZK vs Vehicle	-0.9378 to 0.9378	>0.9999
+KTC	1,25D3 vs Vehicle	0.2971 to 2.463	0.0102
	ZK vs Vehicle	-1.284 to 0.8821	>0.9999
	1,25D3 + ZK vs Vehicle	-1.225 to 0.9408	>0.9999
**s100g**			
	1,25D3 vs Vehicle	0.08666 to 1.276	0.0224
	ZK vs Vehicle	0.04940 to 1.238	0.0317
	1,25D3 + ZK vs Vehicle	-0.8920 to 0.4372	0.7593
+KTC	1,25D3 vs Vehicle	0.2101 to 1.399	0.0069
	ZK vs Vehicle	-0.7141 to 0.4748	0.9365
	1,25D3 + ZK vs Vehicle	-0.5681 to 0.6209	0.9992
**Atp2b1**			
	1,25D3 vs Vehicle	0.1642 to 1.189	0.0081
	ZK vs Vehicle	0.03144 to 1.057	0.0356
	1,25D3 + ZK vs Vehicle	-0.8623 to 0.1628	0.2465
+KTC	1,25D3 vs Vehicle	-0.1989 to 0.8262	0.0315
	ZK vs Vehicle	-0.6352 to 0.3899	0.9014
	1,25D3 + ZK vs Vehicle	-0.3732 to 0.6520	0.8633
**trpv6**			
	1,25D3 vs Vehicle	0.2207 to 1.327	0.0053
	ZK vs Vehicle	0.03403 to 1.141	0.0357
	1,25D3 + ZK vs Vehicle	-0.8910 to 0.3463	0.5953
+KTC	1,25D3 vs Vehicle	0.09541 to 1.202	0.0193
	ZK vs Vehicle	-0.3316 to 0.7751	0.6627
	1,25D3 + ZK vs Vehicle	-0.4452 to 0.6615	0.9415

Figure 1b	Tukey's multiple comparisons test	95% confidence interval	p-value
**2h**			
	1,25D3 vs Vehicle	-1.188 to -0.06841	0.0219
**4h**			
	1,25D3 vs Vehicle	-1.637 to -0.517	<0.0001
**6h**			
	1,25D3 vs Vehicle	-1.96 to -0.6765	<0.0001

Figure 1d	Tukey's multiple comparisons test	95% confidence interval	p-value
**2h**			
	1,25D3 vs Vehicle	-0.2052 to 0.04889	0.1227
	ZK vs Vehicle	-0.2661 to -0.1597	0.0033
	1,25D3 + ZK vs Vehicle	-0.2810 to -0.02973	0.0332
	ZK vs 1,25D3	-0.2209 to -0.04853	0.0211
	1,25D3+ZK vs 1,25D3	-0.3205 to 0.1661	0.35
**4h**			
	1,25D3 vs Vehicle	-0.1990 to -0.05034	0.0183
	ZK vs Vehicle	-0.3393 to -0.1030	0.0148
	1,25D3 + ZK vs Vehicle	-0.2353 to -0.09690	0.0092
	ZK vs 1,25D3	-0.1403 to -0.05269	0.0109
	1,25D3+ZK vs 1,25D3	-0.1850 to 0.1021	0.04061
**6h**			
	1,25D3 vs Vehicle	-0.3043 to -0.1897	0.0018
	ZK vs Vehicle	-0.3173 to -0.2089	0.0009
	1,25D3 + ZK vs Vehicle	-0.4519 to -0.02152	0.0416
	ZK vs 1,25D3	-0.1277 to 0.09547	0.7683
	1,25D3+ZK vs 1,25D3	-0.1494 to 0.1700	0.9644

Figure 1f	Tukey's multiple comparisons test	95% confidence interval	p-value
**FB-789**			
	1,25D3 vs Vehicle	-3.312 to -1.031	0.0023
	ZK vs Vehicle	-2.467 to -0.1855	0.0262
	1,25D3 + ZK vs Vehicle	-1.987 to 0.5117	0.3009
**FB-CYP**			
	1,25D3 vs Vehicle	-3.851 to -0.9949	0.0042
	ZK vs Vehicle	-1.935 to 1.258	>0.9999
	1,25D3 + ZK vs Vehicle	-1.695 to 1.498	>0.9999

Figure 3b	Tukey's multiple comparisons test	95% confidence interval	p-value
**cytosol**			
	1,25D3 vs Vehicle	-0.1343 to 0.4303	0.3771
	1,25D3 + ZK vs Vehicle	-0.4475 to 0.1561	0.4331
	1,25D3 vs 1,25D3 + ZK	-0.5760 to -0.01140	0.0412
**Nucleus**			
	1,25D3 vs Vehicle	-1.215 to -0.4558	0.0002
	1,25D3 + ZK vs Vehicle	-0.6941 to 0.06468	0.1092
	1,25D3 vs 1,25D3 + ZK	0.1411 to 0.8998	0.0085

Figure 3d	Tukey's multiple comparisons test	95% confidence interval	p-value
**cytosol**			
	1,25D3 vs Vehicle	0.1179 to 0.4458	0.0006
	ZK vs Vehicle	-0.09035 to 0.2376	0.732
	1,25D3 + ZK vs Vehicle	-0.05639 to 0.2715	0.3394

**Nucleus**			
	1,25D3 vs Vehicle	-0.5923 to -0.2644	<0.0001
	ZK vs Vehicle	-0.5879 to -0.2600	<0.0001
	1,25D3 + ZK vs Vehicle	-0.3447 to -0.01676	0.0266
	1,25D3 vs 1,25D3 + ZK	0.5481 to 1.892	0.0005

**Figure 3f**	**Tukey's multiple comparisons test**	**95% confidence interval**	**p-value**
**KTC cytosol**			
	1,25D3 vs Vehicle	-0.4506 to 0.6408	0.9582
	ZK vs Vehicle	-0.7415 to 0.3500	0.7369
	1,25D3 + ZK vs Vehicle	-0.6948 to 0.3967	0.8618
**KTC Nucleus**			
	1,25D3 vs Vehicle	-1.215 to -0.1232	0.0139
	ZK vs Vehicle	-0.4380 to 0.6535	0.941
	1,25D3 + ZK vs Vehicle	-0.3535 to 0.7380	0.7471
	1,25D3 vs ZK	0.2310 to 1.322	0.0044
	1,25D3 vs 1,25D3 + ZK	0.3155 to 1.407	0.0018

**Figure 4f**		**Umpaired t test**	**p-value**
	**Cyp24a1**		
		SiWbp4 vs SiControl	0.0319
	**s100g**		
		SiWbp4 vs SiControl	0.0456
	**Atp2b1**		
		SiWbp4 vs SiControl	0.0452

**Figure 4g**	**Tukey's multiple comparisons test**	**95% confidence interval**	**p-value**
**Cyp24a1**			
SiControl	1,25D3 vs Vehicle	1.629 to 10.28	0.0087
	1,25D3 + ZK vs Vehicle	-3.708 to 4.943	0.9219
SiWbp4	1,25D3 vs Vehicle	1.170 to 10.84	0.0163
	1,25D3 + ZK vs Vehicle	0.8583 to 10.53	0.022
**s100g**			
SiControl	1,25D3 vs Vehicle	0.6758 to 6.083	0.0144
	1,25D3 + ZK vs Vehicle	-2.686 to 2.721	0.9998
SiWbp4	1,25D3 vs Vehicle	0.008517 to 5.369	0.0492
	1,25D3 + ZK vs Vehicle	0.4399 to 6.170	0.0234
**Atp2b1**			
SiControl	1,25D3 vs Vehicle	0.4576 to 4.249	0.013
	1,25D3 + ZK vs Vehicle	-1.604 to 2.187	>0.9999
SiWbp4	1,25D3 vs Vehicle	0.08553 to 4.324	0.0401
	1,25D3 + ZK vs Vehicle	0.08012 to 4.319	0.0406

Figure 5a	Tukey's multiple comparisons test	95% confidence interval	p-value
**Calcium**			
	1,25D3 vs Vehicle	0.03564 to 0.1998	0.0057
	1,25D3+ZK 0.3 ug/kg vs Vehicle	-0.05863 to 0.1055	0.8748
	1,25D3 + ZK 1ug/kg vs Vehicle	-0.1296 to 0.03460	0.3743
	VDR-null vs Vehicle	-0.2323 to -0.06813	0.0009
	VDR -/- vs 1,25D3 + ZK 1ug/kg	-0.1848 to -0.02065	0.0139

Figure 5c	Tukey's multiple comparisons test	95% confidence interval	p-value
**Cyp24a1**			
	1,25D3 vs Vehicle	0.5453 to 2.439	0.0208
	1,25D3 + ZK 1ug/kg vs Vehicle	-1.393 to 1.276	0.9643
**Slc30a10**			
	1,25D3 vs Vehicle	0.6763 to 1.576	0.0083
	1,25D3 + ZK 1ug/kg vs Vehicle	-0.3523 to 0.9828	0.1908
**Slc37a2**			
	1,25D3 vs Vehicle	0.7242 to 2.151	0.0128
	1,25D3 + ZK 1ug/kg vs Vehicle	-2.331 to 1.650	0.6418
**Atp2b1**			
	1,25D3 vs Vehicle	0.6757 to 1.940	0.0122
	1,25D3 + ZK 1ug/kg vs Vehicle	-1.283 to 1.082	0.879
**Nkain1**			
	1,25D3 vs Vehicle	0.2554 to 1.589	0.0468
	1,25D3 + ZK 1ug/kg vs Vehicle	-2.105 to 3.704	0.4094

Figure 5e	Tukey's multiple comparisons test	95% confidence interval	p-value
**Slc30a10**	1,25D3 WT vs Vehicle WT	-1.574 to -0.2210	0.0097
	1,25D3+ZK VDR null vs WT 1,25D3+ZK	-1.437 to 0.02488	0.0593
	1,25D3 WT vs 1,25D3+ZK WT	0.2375 to 1.591	0.0086
**Cyp24a1**	1,25D3 WT vs Vehicle WT	-3.806 to -1.383	0.0002
	1,25D3+ZK VDR null vs WT 1,25D3+ZK	0.3449 to 2.962	0.0133
	1,25D3 WT vs 1,25D3+ZK WT	1.341 to 3.764	0.0003
**Slc37a2**	1,25D3 WT vs Vehicle WT	-1.707 to -0.4892	0.001
	1,25D3+ZK VDR null vs WT 1,25D3+ZK	0.8732 to 2.189	0.0001
	1,25D3 WT vs 1,25D3+ZK WT	0.1775 to 1.396	0.0116
**Nkain1**	1,25D3 WT vs Vehicle WT	-1.621 to 0.1988	0.145
	1,25D3+ZK VDR null vs WT 1,25D3+ZK	-1.817 to 0.1494	0.1057
	1,25D3 WT vs 1,25D3+ZK WT	-0.08013 to 1.740	0.0774
**Atp2b1**	1,25D3 WT vs Vehicle WT	-1.617 to -0.4263	0.0032
	1,25D3+ZK VDR null vs WT 1,25D3+ZK	-0.9715 to 0.3595	0.4741
	1,25D3 WT vs 1,25D3+ZK WT	0.9209 to 2.252	0.0004

Figure 6b	Tukey's multiple comparisons test	95% confidence interval	p-value
**Calcium**			
	1,25D3 vs Vehicle	0.06159 to 0.6073	0.0339
	1,25D3 + ZK 1ug/kg vs Vehicle	-0.2255 to 0.4540	0.3173
	1,25D3 vs 1,25D3 + ZK 1ug/kg	-0.3580 to -0.08246	0.0202

Figure 6c	Tukey's multiple comparisons test	95% confidence interval	p-value
**PTH**			
	1,25D3 vs Vehicle	-2.167 to -1.326	0.0024
	1,25D3 + ZK 1ug/kg vs Vehicle	-2.154 to 1.684	0.7772
	1,25D3 vs 1,25D3 + ZK 1ug/kg	0.004253 to 3.018	0.0497

Figure 6d	Tukey's multiple comparisons test	95% confidence interval	p-value
**Duodenal**			
	1,25D3 vs Vehicle	0.06159 to 0.6073	0.0339
	1,25D3 + ZK 1ug/kg vs Vehicle	-0.2255 to 0.4540	0.3173
	1,25D3 vs 1,25D3 + ZK 1ug/kg	-0.3580 to -0.08246	0.0202

	Tukey's multiple comparisons test	95% confidence interval	p-value
**Renal**			
	1,25D3 vs Vehicle	0.2821 to 1.279	0.0071
	1,25D3 + ZK 1ug/kg vs Vehicle	-0.6336 to 0.3637	0.6995
	1,25D3 vs 1,25D3 + ZK 1ug/kg	-1.414 to -0.4170	0.0032

Sup Fig 3a	Tukey's multiple comparisons test	95% confidence interval	p-value
**Wbp4**			
SiControl	1,25D3 vs Vehicle	-1.473 to 2.275	0.8498
	1,25D3 + ZK vs Vehicle	-2.795 to 0.9540	0.4386
SiWbp4	1,25D3 vs Vehicle	-2.809 to 0.9395	0.4278
	1,25D3 + ZK vs Vehicle	-2.662 to 1.087	0.5427

Sup Fig 3d	Tukey's multiple comparisons test	95% confidence interval	p-value
**Wbp4**			
	SiWBP4 Cyt vs SiControl Cyt	-0.7014 to -0.3090	<0.0001
	SiWBP4 Nucl vs SiControl Nucl	-0.5217 to -0.1055	0.0038

Sup Fig 3f	Tukey's multiple comparisons test	95% confidence interval	p-value
**VDR**			
	SiWBP4 Cyt vs SiControl Cyt	0.3739 to 1.368	0.041
	SiWBP4 Nucl vs SiControl Nucl	-1.105 to -0.02279	0.0014
