# Supplementary material for: Cytosolic sequestration of the vitamin D receptor as a therapeutic option for vitamin D-induced hypercalcemia
Source: Nat Commun. 2020 Dec 7;11:6249. doi: 10.1038/s41467-020-20069-4 (PMC7721737; doi:10.1038/s41467-020-20069-4)
Supplement: Supplementary file 3 — Reporting Summary [file 41467_2020_20069_MOESM3_ESM.pdf]

nature research

Corresponding author(s): D. Metzger and G. Laverny

Last updated by author(s): Oct 21, 2020

Reporting Summary

Nature Research wishes to improve the reproducibility of the work that we publish. This form provides structure for consistency and transparency in reporting. For further information on Nature Research policies, see Authors & Referees and the Editorial Policy Checklist .

Statistics

For all statistical analyses, confirm that the following items are present in the figure legend, table legend, main text, or Methods section.

n/a	Confirmed	
☐	☒	The exact sample size (*n*) for each experimental group/condition, given as a discrete number and unit of measurement
☐	☒	A statement on whether measurements were taken from distinct samples or whether the same sample was measured repeatedly
☐	☒	The statistical test(s) used AND whether they are one- or two-sided<br>*Only common tests should be described solely by name; describe more complex techniques in the Methods section.*
☐	☒	A description of all covariates tested
☒	☐	A description of any assumptions or corrections, such as tests of normality and adjustment for multiple comparisons
☐	☒	A full description of the statistical parameters including central tendency (e.g. means) or other basic estimates (e.g. regression coefficient) AND variation (e.g. standard deviation) or associated estimates of uncertainty (e.g. confidence intervals)
☐	☒	For null hypothesis testing, the test statistic (e.g. *F*, *t*, *r*) with confidence intervals, effect sizes, degrees of freedom and *P* value noted<br>*Give P values as exact values whenever suitable.*
☒	☐	For Bayesian analysis, information on the choice of priors and Markov chain Monte Carlo settings
☒	☐	For hierarchical and complex designs, identification of the appropriate level for tests and full reporting of outcomes
☒	☐	Estimates of effect sizes (e.g. Cohen's *d*, Pearson's *r*), indicating how they were calculated

Our web collection on statistics for biologists contains articles on many of the points above.

Software and code

Policy information about availability of computer code

Data collection	Hiseq 4000 sequencer (Illumina)<br>RTA 2.7.7, bcl2fastq 2.17.1.14<br>Leica SP8 UV/Visible Laser Confocal Microscope equipped with a Leica HC PL APO 63x 1.4N.A. oil immersion objective<br>ImageQuant LAS 4000 biomolecular imager (GE Healthcare)<br>Monolith NT.115 instrument (NanoTemper Technologies GmbH)
Data analysis	Data analyses were performed with GraphPad Software Prism 8 (version 8.03).<br>For RNA-seq, mass spectrometry, Microscale thermophoresis and HDX, our data were processed as described in material and methods section.<br>Images were analyzed with FIJI/ ImageJ distribution Software v1.53c (https://imagej.nih.gov/ij/).

For manuscripts utilizing custom algorithms or software that are central to the research but not yet described in published literature, software must be made available to editors/reviewers. We strongly encourage code deposition in a community repository (e.g. GitHub). See the Nature Research guidelines for submitting code & software for further information.

Data

Policy information about availability of data

All manuscripts must include a data availability statement. This statement should provide the following information, where applicable:

- Accession codes, unique identifiers, or web links for publicly available datasets
- A list of figures that have associated raw data
- A description of any restrictions on data availability

RNA sequencing raw data are available in GEO database [(GSE141985) https://www.ncbi.nlm.nih.gov/geo/query/acc.cgi?acc=GSE141985)]. Mass spectrometry raw data of IEC-18 cells are available as a Source Data file Fig. 3a. HDX-MS raw data are available via ProteomeXchange with identifier PXD019810 (http://proteomecentral.proteomexchange.org/cgi/GetDataset?ID=PXD019810). The source data underlying Figs 1-6 and Extended Data Fig 1-6 are provided as Source

Data files.

Field-specific reporting

Please select the one below that is the best fit for your research. If you are not sure, read the appropriate sections before making your selection.

☒ Life sciences ☐ Behavioural & social sciences ☐ Ecological, evolutionary & environmental sciences

For a reference copy of the document with all sections, see nature.com/documents/nr-reporting-summary-flat.pdf

Life sciences study design

All studies must disclose on these points even when the disclosure is negative.

Sample size	As the expected effects were between 20 - 40 %, we estimated from our previous studies that 3 animals per group will provide sufficient statistical power (Journal of Medicinal Chemistry 2009, 52, 8, 2204-2213). Sample size are clearly stated in the figure legends.
Data exclusions	No inclusion/exclusion criteria was used in this study.
Replication	All experiments were reliably reproducible at least in 3 biological independent experiments.
Randomization	All samples used in the study were randomly allocated into different experimental groups.
Blinding	In vitro and in vivo experiments were not blinded as were designed and carried out by the first author. Use of well established softwares and pipeline reduced the bias.

Reporting for specific materials, systems and methods

We require information from authors about some types of materials, experimental systems and methods used in many studies. Here, indicate whether each material, system or method listed is relevant to your study. If you are not sure if a list item applies to your research, read the appropriate section before selecting a response.

Materials & experimental systems

n/a	Involved in the study	
☐	☒	Antibodies
☐	☒	Eukaryotic cell lines
☒	☐	Palaeontology
☐	☒	Animals and other organisms
☐	☒	Human research participants
☒	☐	Clinical data

Methods

n/a	Involved in the study	
☒	☐	ChIP-seq
☒	☐	Flow cytometry
☒	☐	MRI-based neuroimaging

Antibodies

Antibodies used	The list of the antibodies used for this study is available in Extended table 3.

Validation	Primary antibodies.<br>VDR (ref 12550) No signal detected in VDR-null mice (see Extended data Fig. 4). Peer-reviewed citations at https://www.cellsignal.com/products/primary-antibodies/vitamin-d3-receptor-d2k6w-rabbit-mab/12550.<br>WBP4 (ab 108144). Lower signal detected in silencing experiments (see Extended data Fig. 3). Peer-reviewed citations at https://www.abcam.com/ww-domain-binding-protein-4-antibody-ab108144.html.<br>WBP4 (ab 272629). Lower signal detected in silencing experiments (see Extended data Fig. 3). Peer-reviewed citations at https://www.abcam.com/ww-domain-binding-protein-4-antibody-ab272629.html<br>Lamin B1 (ab16048). No signal in cytosolic protein extracts (Fig. 2). Peer-reviewed citations at https://www.abcam.com/lamin-b1-antibody-nuclear-envelope-marker-ab16048.html.<br>Gapdh (ref 2118). Peer-reviewed citations at https://www.cellsignal.com/products/primary-antibodies/gapdh-14c10-rabbit-mab/2118.<br>Secondary antibodies.<br>111-035-003. Peer-reviewed citations at https://www.jacksonimmuno.com/catalog/products/111-035-003.<br>111-165-003. Peer-reviewed citations at https://www.jacksonimmuno.com/catalog/products/111-165-003.<br>115-035-003. Peer-reviewed citations at https://www.jacksonimmuno.com/catalog/products/115-035-003.<br>ref 3678. Peer-reviewed citations at https://www.cellsignal.com/products/secondary-antibodies/mouse-anti-rabbit-igg-conformation-specific-l27a9-mab/3678.<br>C15410206. Peer-reviewed citations at https://www.diagenode.com/en/p/rabbit-igg-250-ug-250-ul.

Eukaryotic cell lines

Policy information about cell lines

Cell line source(s)	IEC-18 were purchased from ATCC. FB-789, U2OS and HeLa cells were obtained from IGBMC cell culture facility.
Authentication	NO cell line authentication was performed.
Mycoplasma contamination	All the cell lines were tested negative for mycoplasma contamination.
Commonly misidentified lines (See ICLAC register)	No commonly misidentified cell lines were used in the study.

Animals and other organisms

Policy information about studies involving animals; ARRIVE guidelines recommended for reporting animal research

Laboratory animals	Ten week-old C57BL/6J male mice. Ten week-old VDR-null (VDR-/-) male mice on a similar genetic background were used.
Wild animals	NO Wild animals were used in this study.
Field-collected samples	NO field-collected samples were used in this study.
Ethics oversight	All animal experimental protocols were conducted in compliance with French and EU regulations on the use of laboratory animals for research, and approved by the IGBMC Ethical Committee and the French Ministry (#10047-2017052615101492 and #21776-2019082318288737).

Note that full information on the approval of the study protocol must also be provided in the manuscript.

Human research participants

Policy information about studies involving human research participants

Population characteristics	*Describe the covariate-relevant population characteristics of the human research participants (e.g. age, gender, genotypic information, past and current diagnosis and treatment categories). If you filled out the behavioural & social sciences study design questions and have nothing to add here, write "See above."*
Recruitment	*Describe how participants were recruited. Outline any potential self-selection bias or other biases that may be present and how these are likely to impact results.*
Ethics oversight	*Identify the organization(s) that approved the study protocol.*

Note that full information on the approval of the study protocol must also be provided in the manuscript.
